# Water immersion decreases sympathetic skin response during color–word Stroop test

**DOI:** 10.1371/journal.pone.0180765

**Published:** 2017-07-24

**Authors:** Daisuke Sato, Yudai Yamazaki, Akari Takahashi, Yoshihito Uetake, Saki Nakano, Kaho Iguchi, Yasuhiro Baba, Rio Nara, Yoshimitsu Shimoyama

**Affiliations:** 1 Institute for Human Movement and Medical Sciences, Niigata University of Health and Welfare, Niigata city, Niigata, Japan; 2 Graduate school for Major in Health Science, Niigata University of Health and Welfare, Niigata city, Niigata, Japan; 3 Department of Health and Sports, Niigata University of Health and Welfare, Niigata city, Niigata, Japan; Westmead Millenium Institute, AUSTRALIA

## Abstract

Water immersion alters the autonomic nervous system (ANS) response in humans. The effect of water immersion on executive function and ANS responses related to executive function tasks was unknown. Therefore, this study aimed to determine whether water immersion alters ANS response during executive tasks. Fourteen healthy participants performed color–word-matching Stroop tasks before and after non-immersion and water immersion intervention for 15 min in separate sessions. The Stroop task-related skin conductance response (SCR) was measured during every task. In addition, the skin conductance level (SCL) and electrocardiograph signals were measured over the course of the experimental procedure. The main findings of the present study were as follows: 1) water immersion decreased the executive task-related sympathetic nervous response, but did not affect executive function as evaluated by Stroop tasks, and 2) decreased SCL induced by water immersion was maintained for at least 15 min after water immersion. In conclusion, the present results suggest that water immersion decreases the sympathetic skin response during the color–word Stroop test without altering executive performance.

## Introduction

Water immersion induces various physiological responses depending on physical parameters such as temperature and hydrostatic pressure [1–3]. These physiological changes can have therapeutic benefits, and water immersion is a part of the rehabilitation regimens for patients with orthopedic, cardiovascular, and respiratory disorders [4, 5]. Previous studies have reported that water immersion alters the autonomic nervous system (ANS) response in humans. Water immersion at a thermoneutral temperature was reported to suppress levels of the fluid-regulating hormones renin, angiotensin II, aldosterone, and arginine vasopressin to normalize blood volume with the increasing cardiac output and stroke volume that occurs during water immersion [6, 7]. In addition, several short-duration (5–30 min) water immersion protocols with different water exposures and postures (resting head out in an upright positon [8], 60° upright position [9], or complete water immersion in an upright position [10]) all increased heart rate variability (HRV). Moreover, the greatest impact of water immersion on HRV is on the high-frequency (HF) component, indicating a shift toward enhanced parasympathetic nervous activity. Interestingly, our recent results using functional near infrared spectroscopy show that oxyhemoglobin concentration in the frontal lobe decreased even after short-term water immersion; an effect which likely involved ANS activity [11, 12] because sympathetic nervous activity is positively correlated with the oxyhemoglobin concentration [13].

Numerous studies have reported that ANS activity is related to performance on executive tasks [14–16]. Executive tasks elicit cardiac acceleration (increased heart rate or reduced heart period), which is mediated by increased sympathetic and decreased parasympathetic cardiac activity [17, 18]. Mathewson et al. [19] investigated predicted relations between ANS activity and executive function during a Stroop task and suggested that engagement in Stroop tasks appeared to elicit increased sympathetic activity in order to support neural processing required for task performance. The authors further reported that a moderate increase in sympathetic activity during task performance represents an appropriate adaptation to mild stress. Moreover, greater task difficulty involved increased sympathetic activity in carrying out executive tasks [16]. Based on these results, it is possible that decreased sympathetic activity induced by water immersion influences performance on executive tasks. Thayer et al. [15] proposed that the relationship between executive performance and HRV is related to the common neural basis of both functions. In addition, Lane et al. [20] reported that the medial visceromotor network is a terminal pathway by which emotional and executive functions recruit autonomic support.

To date, no study has examined the effects of water immersion on ANS activity during tasks requiring executive function. We hypothesized that ANS activity induced by water immersion decreases sympathetic nervous system activity during executive tasks and decreases executive function performance. If our hypothesis were proven true, water immersion and exercise should be carefully used because decreased executive function is strongly related to falls in the elderly [21]. Therefore, the aim of the present study was to examine whether water immersion alters ANS performance during executive tasks.

## Materials and methods

### Participants and experimental design

We examined 14 healthy adults (7 male and 7 female; aged 19–24 years), all of whom provided informed consent. They were also asked to refrain from ingesting beverages containing caffeine and alcohol and not to exercise during the 24 h preceding each experiment. All subjects were right handed, and none had a history of neurological or psychiatric disease or were taking any medication. The present study was conducted in accordance with the tenets of the Declaration of Helsinki and approved by the ethical committee of the Niigata University of Health and Welfare. Table 1 shows physiological characteristics of all participants.

**Table 1:** Physiological characteristics of all participants.

Participant	Sex	Age (y.o.)	Height (cm)	Weight (kg)	Body mass index
1	F	19	168.7	57.5	20.2
2	F	19	168.3	58.7	20.7
3	F	20	162.7	55.6	21.0
4	F	21	164.0	59.0	21.9
5	F	21	167.0	60.3	21.6
6	M	20	175.6	67.2	21.8
7	M	21	175.5	69.3	22.5
8	M	21	173.9	62.1	20.5
9	M	21	173.5	64.7	21.5
10	M	22	171.0	62.5	21.4
11	M	23	178.0	73.0	23.0
12	F	22	167.0	58.2	20.9
13	F	21	158.0	53.0	21.2
14	M	24	170.0	57.0	19.7
Average		21.1	169.5	61.3	21.3
SE		0.4	1.5	1.5	0.2

**Fig 1:**
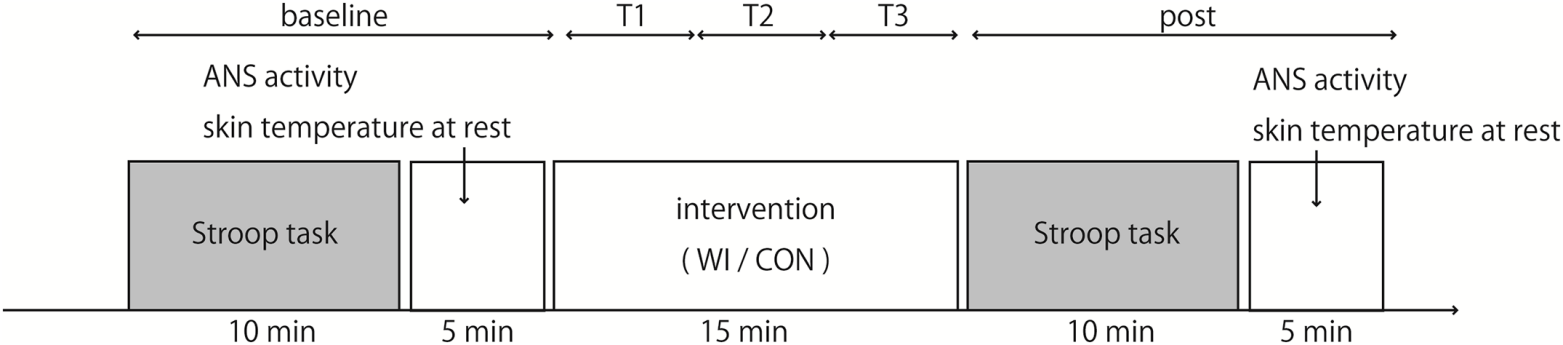
Schematic of the experimental protocol.

The subjects wore only swimwear and were seated on a comfortable reclining armchair with a mounted headrest during measurement and intervention. The experimental sequence included baseline assessment (baseline), assessment at 3 time points during the 15-min intervention (T1, T2, and T3), and assessment after intervention (post) (Fig 1). Baseline and post-assessments included autonomic activity and skin temperature measurement at rest and executive task-induced sympathetic nervous activity. This setup was performed using 2 separate protocols in a randomized order: (1) non-immersed control (CON) and (2) water-immersed intervention (WI). All participants underwent the experimental protocol at an interval of at least 5 days between sessions at the same time of the day (between 10:00 and 16:00 hours).

### Intervention

Each intervention (CON and WI) lasted 15 min. The subjects were seated on a reclining armchair at rest in an empty tank (CON) or in water (WI). The subjects assumed the same body position in both interventions using a belt to avoid muscle contractions. For WI, ambient and water temperatures were set at 28°C ± 1°C and 34°C ± 1°C, respectively. The tank in which the participants were seated was filled with water up to the axillary level of each participant, and the left hand was placed on a table above the water level for skin conductance level (SCL) recording. For CON intervention, ambient temperature was set at 28°C ± 1°C, and participants were seated on a reclining armchair in the same tank as WI but without water.

### Executive function (Stroop task)

**Fig 2:**
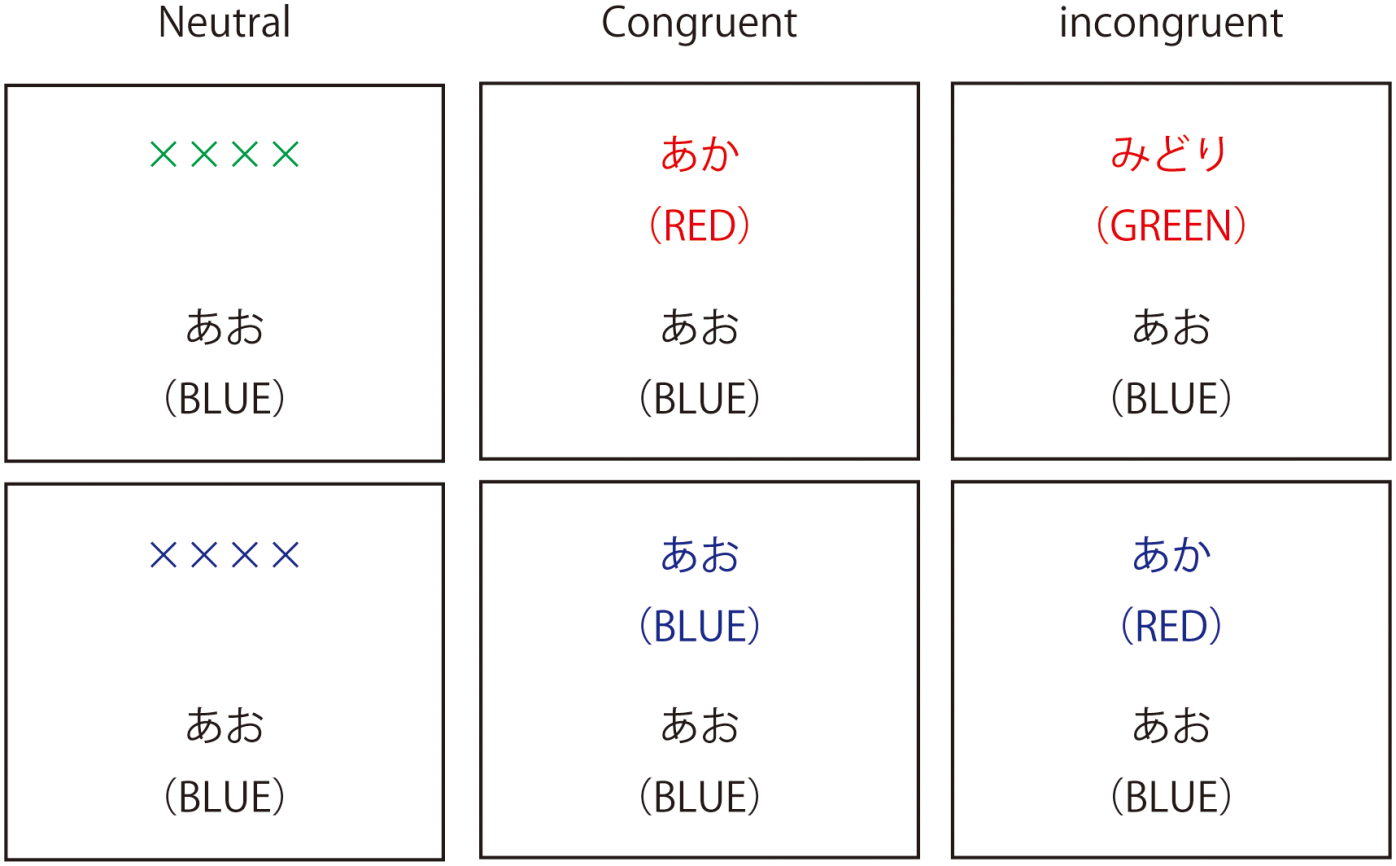
Color–word-matching Stroop task. Instances of single trials for the neutral, congruent, and incongruent events of the color–word- matching Stroop task are depicted. Stimuli were presented in Japanese. Their English translations are indicated in parentheses. The question asked (in Japanese) was as follows: “Does the color of the upper word match the meaning of the lower word?” For the top three examples, the correct answer was “No,” and for the bottom three examples, the correct answer was “Yes.” Stroop task performance was evaluated and recorded using a multimodality encoder system (Super Lab X5, Cedrus, CA).

We adopted the color–word-matching Stroop task [22–24] in an event-related design. We presented two rows of letters on a computer screen and instructed the subjects to decide whether the color of the letters in the top row corresponded to the name of the color printed in the bottom row; furthermore, we asked the subjects to respond by pressing buttons to provide “Yes” or “No” responses with their middle fingers (Fig 2). The order of the two buttons was changed so that for half the total number of the subjects, the “Yes” button was on the left, and for the other half, it was on the right. Correct answer rate and reaction time and error rate were also measured. For neutral trials, the top row contained groups of X’s (XXX) printed in red, green, blue, or yellow and the bottom row contained the words “RED,” “GREEN,” “BLUE,” and “YELLOW” printed in black. For congruent trials, the top row contained the words “RED,” “GREEN,” “BLUE,” and “YELLOW” printed in a congruent color. For incongruent conditions, the name of the color name was printed in an incongruent color. All word stimuli were presented in Japanese. The top row was presented 100 ms before the lower row to achieve sequential visual attention [22]. The correct answer rate assigned to yes and no was 50% for each. Each experimental session comprised 30 trials, comprising 10 neutral, 10 congruent, and 10 incongruent events, presented in a random order with an inter-stimulus interval during which a blank screen was shown for 20 s. The stimulus remained on the screen until the response was given or for 2 s. All words were written in Japanese. Prior to the experiment, three practice sessions were performed.

In the present study, Stroop interference was adopted to elucidate the effect of water immersion on executive function, which is a specifically defined cognitive process. During the incongruent events, the two conflicting sources of color information cause a competing effect known as Stroop interference, which is most typically observed as a prolonged reaction time compared to the neutral or congruent events [25]. Therefore, the (incongruent–neutral) contrast, which is assumed to represent Stroop interference, was calculated in accordance with previous studies examining Stroop interference [26, 27].

### SCL and skin conductance response (SCR)

SCL and SCR were recorded using 7.5-mm diameter Ag/AgCl electrodes on the left middle and fourth fingers, with an electrolyte (0.05 M NaCl) in an inert viscous ointment base. A constant voltage device (Model 2701 SC for SCL/SCR data collection system; UFI, Morro Bay, CA) set at 0.5 V was used to record the electrodermal data [28]. SCL is often used in psychophysiology as an objective measure of the level of arousal [29], and as a tonic measure that indicates relatively slow fluctuations of bodily states of arousal during emotional, cognitive, and physical behavior, which are expressed in the activity of sympathetic cholinergic neurons at the level of the eccrine dermal sweat glands [30]. As a measure of arousal level throughout the presently described experiment, the SCLs were averaged every 5 min during both interventions (T1, T2, and T3 during the 15-min intervention) and at baseline and post-assessment. SCR is a rapid, brief phasic electrodermal response to a single stimulus, which can be considered to be superimposed upon the SCL. In the present study, the continuous data were segmented into epochs of 20 s that included a 2-s period before presenting each cognitive task. All epoch data were averaged. The peak amplitude of the SCR was referenced to the baseline level.

### Electrocardiography (ECG)

The ECG signals were recorded continuously using a portable device (BioAmp ML132, AD Instrument, Sydney). The ECG signal was sampled at 1000 Hz and the distribution of R-R intervals and ectopic beats were identified using the HRV analysis module (LabChart, AD Instruments, Sydney) every 5 min during both interventions (T1, T2, and T3) and at baseline and post-assessment. Power spectral analysis was conducted by the same software. The spectrum power was separated by the low frequency (LF) power and HF power. LF has been identified as the frequency band from 0.04 Hz to 0.15 Hz, and HF as the frequency band from 0.15 Hz to 0.40 Hz. Because of distributional violations, these spectral-power estimates were natural logged and then taken as an indicator of cardiac parasympathetic activity. LF/HF ratio estimates were then used as an indicator of sympathovagal balance [31].

### Visual analog scale (VAS)

A 10-cm VAS was used to measure the comfortable/uncomfortable sensation during both interventions and at baseline and post-assessment: −5 indicated extremely uncomfortable and +5 indicated extremely comfortable. Subjects were instructed to draw a vertical line at the appropriate point on the horizontal VAS line to indicate their score.

### Data analysis

Statistical analyses were performed using the SPSS Statistical Package (SPSS Inc., Chicago, IL). For the SCL, VAS, and ECG data, a two-factorial repeated measures analysis of variance (ANOVA) was conducted for within-subject factors time (5 levels: baseline; post-assessment; and T1, T2, and T3) and intervention (2 levels: CON and WI). For the reaction time, Stroop inference time and SCR, a two-factorial repeated measures ANOVA was conducted for within-subject factors time (2 levels: baseline and post-assessment) and intervention (2 levels: CON and WI). If the assumption of sphericity was violated in the Mauchly's sphericity test, the degree of freedom was corrected using Greenhouse–Geisser's correction coefficient epsilon, and the F- and P-values were recalculated. Post hoc tests (Bonferroni–Dunn) were performed, and the significance level was set at 5%. Power analyses indicated that 14 total participants would be needed to detect an effect size f of 0.4, with two-sided alpha set at 0.05 and power at 0.95.

## Results

### Executive function (color–word-matching Stroop task)

Table 2 presents the change in reaction time, Stroop interference time, and error rate of color–word-matching Stroop task. For reaction time, the repeated measures ANOVA revealed no reliable interaction of “intervention” and “time” for the neutral event (F(1,13) = 1.031, p = 0.33), incongruent event (F(1,13) = 0.471, p = 0.51), and Stroop interference time (F(1,13) = 1.032, p = 0.33). There was no significant main effect of “intervention” for the neutral event (F(1,13) = 1.522, p = 0.24), incongruent event (F(1,13) = 3.745, p = 0.08), and Stroop interference time (F(1,13) = 0.421, p = 0.53). In addition, there was no significant main effect of “time” for the neutral event (F(1,13) = 0.157, p = 0.70), incongruent event (F(1,13) = 0.871, p = 0.37), and Stroop interference time (F(1,13) = 0.613, p = 0.45).

**Table 2:** The change in reaction time, Stroop interference time, and error rate of color–word-matching Stroop task.

	CON		WI	
	baseline	post	baseline	post
Reaction time (msec)				
Neutral event	801.8 ± 31.3	810.2 ± 38.5	791.1 ± 41.1	767.8 ± 27.7
Incongruent event	936.3 ± 52.8	934.6 ± 47.6	889.9 ± 56.8	835.1 ± 78.6
Stroop interference time (msec)	134.5 ± 34.9	124.8 ± 35.5	98.8 ± 22.3	138.3 ± 42.0
Error rate (%)				
Neutral event	11.4 ± 4.3	12.1 ± 4.9	10.0 ± 3.5	10.7 ± 4.4
Incongruent event	10.7 ± 1.3	4.3 ± 1.7	15.0 ± 2.9	10.0 ± 1.8

For error rate, repeated measures ANOVA revealed no reliable interaction of “intervention” and “time” in the neutral (F(1,13) = 0.090, p = 0.78) and incongruent (F(1,13) = 0.157, p = 0.70) events. There was no significant main effect of “intervention” (F(1,13) = 1.156, p = 0.30) and “time” (F(1,13) = 0.171, p = 0.69) for the neutral event. However, there was a significant main effect of “intervention” (F(1,13) = 5.353, p = 0.038) and “time” (F(1,13) = 8.000, p = 0.014) for the incongruent event. Post hoc comparisons revealed that there were significant changes between baseline and post-assessment in the CON intervention and WI in congruent events (p = 0.014, Bonferroni corrected).

### Stroop task- and interference-related SCR

**Fig 3:**
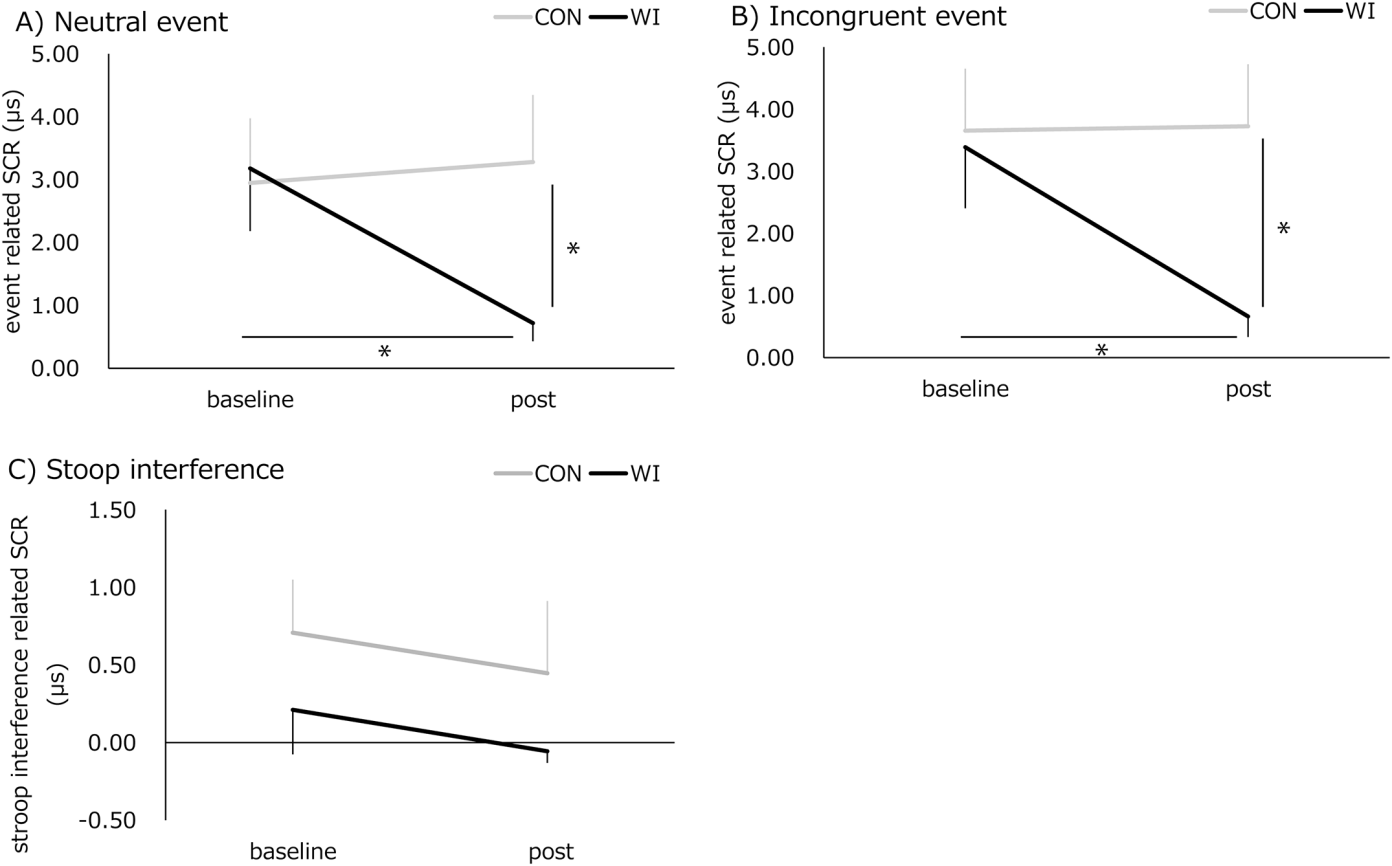
The change in Stroop task- and interference-related skin conductance responses. A) Neutral event-related SCR, B) Incongruent event-related SCR, C) Stroop interference-related SCR. *; significant difference (p < 0.05).

Fig 3 and Table 3 show the change in Stroop task- and Stroop interference-related SCR. The repeated measures ANOVA revealed reliable interactions of “intervention” and “time” for the neutral (F(1,13) = 8.252, p = 0.013) and incongruent (F(1,13) = 13.033, p = 0.003) events. There was no significant main effect of “intervention” and “time” for the neutral events (F(1,13) = 3.871, p = 0.07 and F(1,13) = 4.257, p = 0.06, respectively). There was a significant main effect of “time” (F(1,13) = 10.275, p = 0.007) for the incongruent events, but there was no main effect of “intervention” (F(1,13) = 4.066, p = 0.07). Post hoc comparisons revealed significantly lower event-related SCR for WI compared with CON at post-assessment (neutral event: p = 0.017, incongruent event: p = 0.009). Additionally, we found WI significantly decreased event-related SCR at post-assessment compared with baseline for both neutral (p = 0.010) and incongruent events (p = 0.003). Repeated measures ANOVA revealed no significant interaction of “intervention” and “time” (F(1,13) = 0.001, p = 0.99) and no main effect of either “intervention” (F(1,13) = 1.964, p = 0.18) or “time” (F(1,13) = 0.389, p = 0.54) in the Stroop inference-related SCR.

**Table 3:** The change in Stroop task- and Stroop interference-related SCR.

		CON		WI	
		baseline	post	baseline	post
Stroop task-related SCR					
Neutral event	(μs)	2.9 ± 1.0	3.3 ± 1.1	3.2 ± 1.0	0.7 ± 0.3
Incongruent event	(μs)	3.7 ± 1.2	3.7 ± 1.1	3.4 ± 1.0	0.7 ± 0.3
Stroop interference-related SCR	(μs)	0.7 ± 0.3	0.4 ± 0.5	0.2 ± 0.3	-0.1 ± 0.1

### SCL

**Fig 4:**
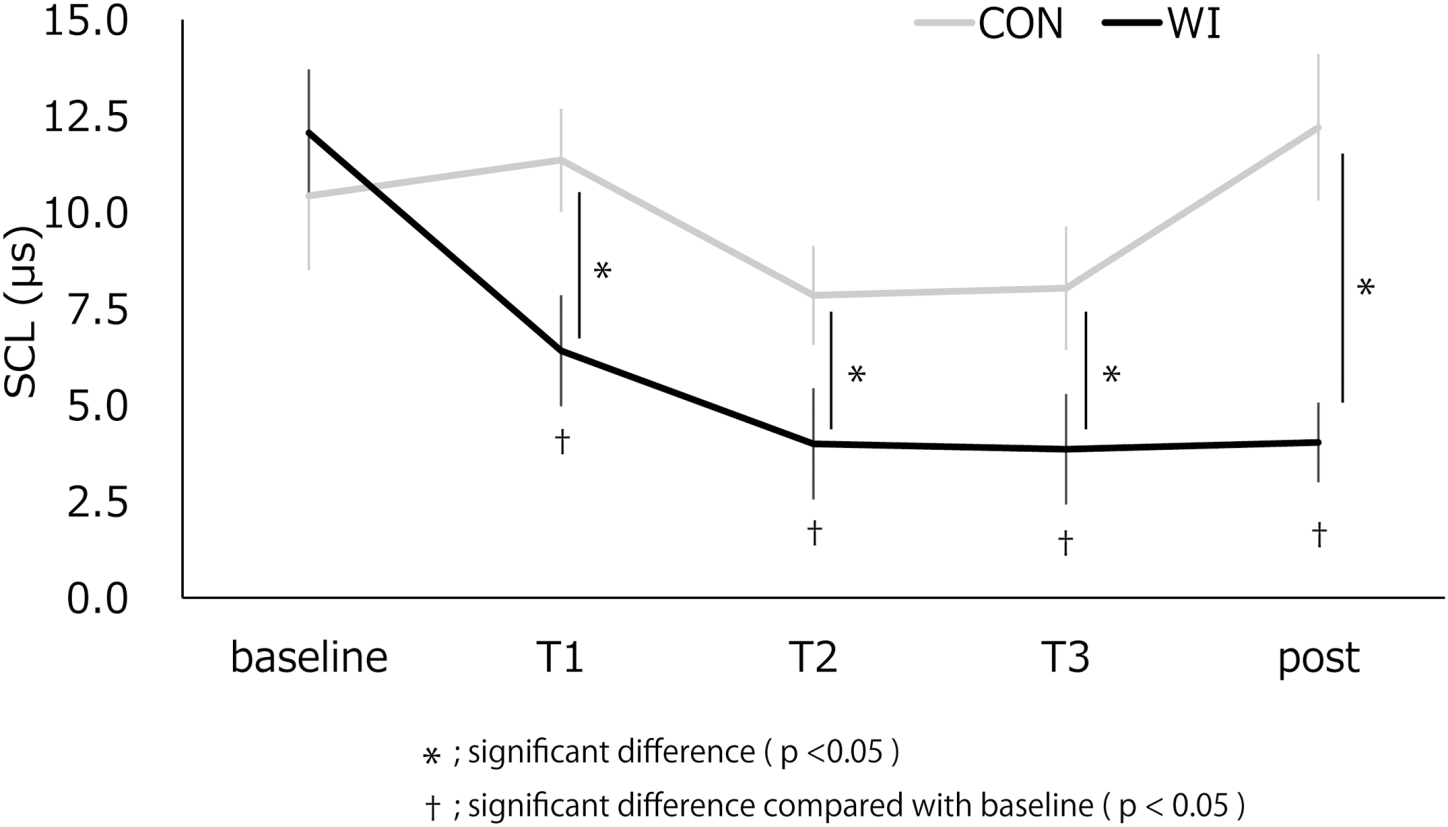
The change in skin conductance level at baseline, T1, T2, T3, and post-assessment. *; significant difference (p <0.05) †; significant difference compared with baseline (p < 0.05).

Fig 4 and Table 4 show the change in SCL. The repeated measures ANOVA revealed a reliable interaction of “intervention” and “time” (F(4,52) = 9.404, p = 0.000). Besides this interaction, significant main effects of “intervention” (F(1,13) = 6.831, p = 0.021) and “time” (F(4,52) = 7.986, p = 0.000) were revealed. Post hoc comparisons revealed significantly lower SCL in WI than in CON at T1, T2, T3, and post-assessment (p = 0.012, 0.047, 0.049, 0.002, respectively). Furthermore, for WI only, we found that SCL significantly decreased at T1, T2, T3, and post-assessment compared with baseline (p = 0.049, 0.010, 0.014, 0.003, respectively).

**Table 4:** Changes in SCL, HF component, LF/HF ratio and VAS scale.

	CON	WI
SCL (μs)		
baseline	10.42 ± 1.92	12.06 ± 1.64
T1	11.35 ± 1.34	6.41 ± 1.44
T2	7.85 ± 1.28	3.99 ± 1.44
T3	8.03 ± 1.60	3.86 ± 1.43
post	12.20 ± 1.90	4.03 ± 1.03
HF component (msec^2^)		
baseline	310.60 ± 71.12	259.95 ± 65.39
T1	396.79 ± 121.28	1025.20 ± 228.73
T2	413.65 ± 146.05	933.40 ± 249.36
T3	353.28 ± 134.61	1052.20 ± 253.68
post	464.21 ± 246.54	402.56 ± 120.08
LF/HF ratio		
baseline	0.97 ± 0.14	1.22 ± 0.20
T1	1.44 ± 0.24	0.46 ± 0.09
T2	1.30 ± 0.25	0.40 ± 0.08
T3	1.44 ± 0.27	0.57 ± 0.10
post	1.31 ± 0.24	1.09 ± 0.22
VAS		
baseline	0.13 ± 0.18	-0.05 ± 0.10
T1	0.38 ± 0.29	0.71 ± 0.38
T2	0.69 ± 0.29	1.59 ± 0.43
T3	0.52 ± 0.40	1.92 ± 0.52
post	0.31 ± 0.61	0.01 ± 0.43

### HF and LF/HF

**Fig 5:**
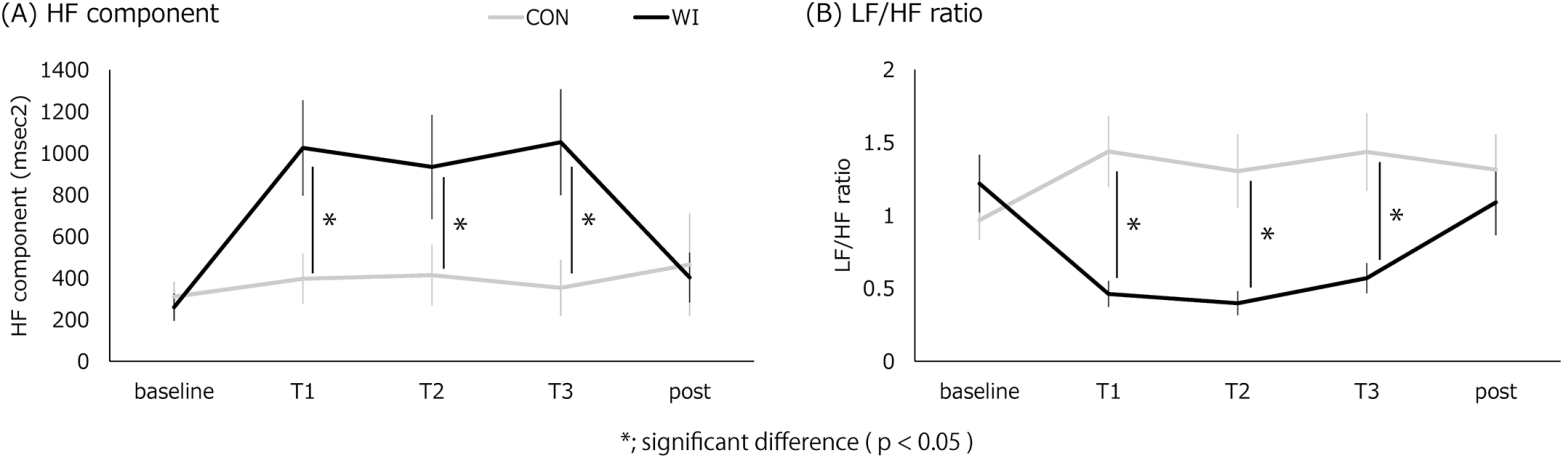
The change in cardiac parasympathetic nervous activity and sympathovagal balance. A) HF component, B) LF/HF ratio. *; significant difference (p < 0.05).

Fig 5(A) and Table 4 present the HF component throughout the experiment. A repeated measures ANOVA revealed a reliable interaction of “intervention” and “time” (F(4,52) = 7.033, p = 0.000). In addition to this interaction, significant main effects of “intervention” (F(1,13) = 6.332, p = 0.026) and “time” (F(4,52) = 4.304, p = 0.004) were revealed. Post hoc comparisons revealed significantly higher values at T1, T2, and T3 in WI than in CON (p = 0.006, 0.025, 0.011, respectively).

Fig 5(B) and Table 4 present the change in the LF/HF ratio. A repeated measures ANOVA revealed a reliable interaction of “intervention” and “time” (F(4,52) = 10.071, p = 0.000). In addition to this interaction, significant main effects of “intervention” (F(1,13) = 10.891, p = 0.006) were revealed, whereas there was no main effect of “time” (F(4,52) = 1.115, p = 0.36). Post hoc comparisons revealed significantly lower values at T1, T2, and T3 in WI than in CON intervention (p = 0.000, 0.002, 0.010, Bonferroni corrected).

### VAS

**Fig 6:**
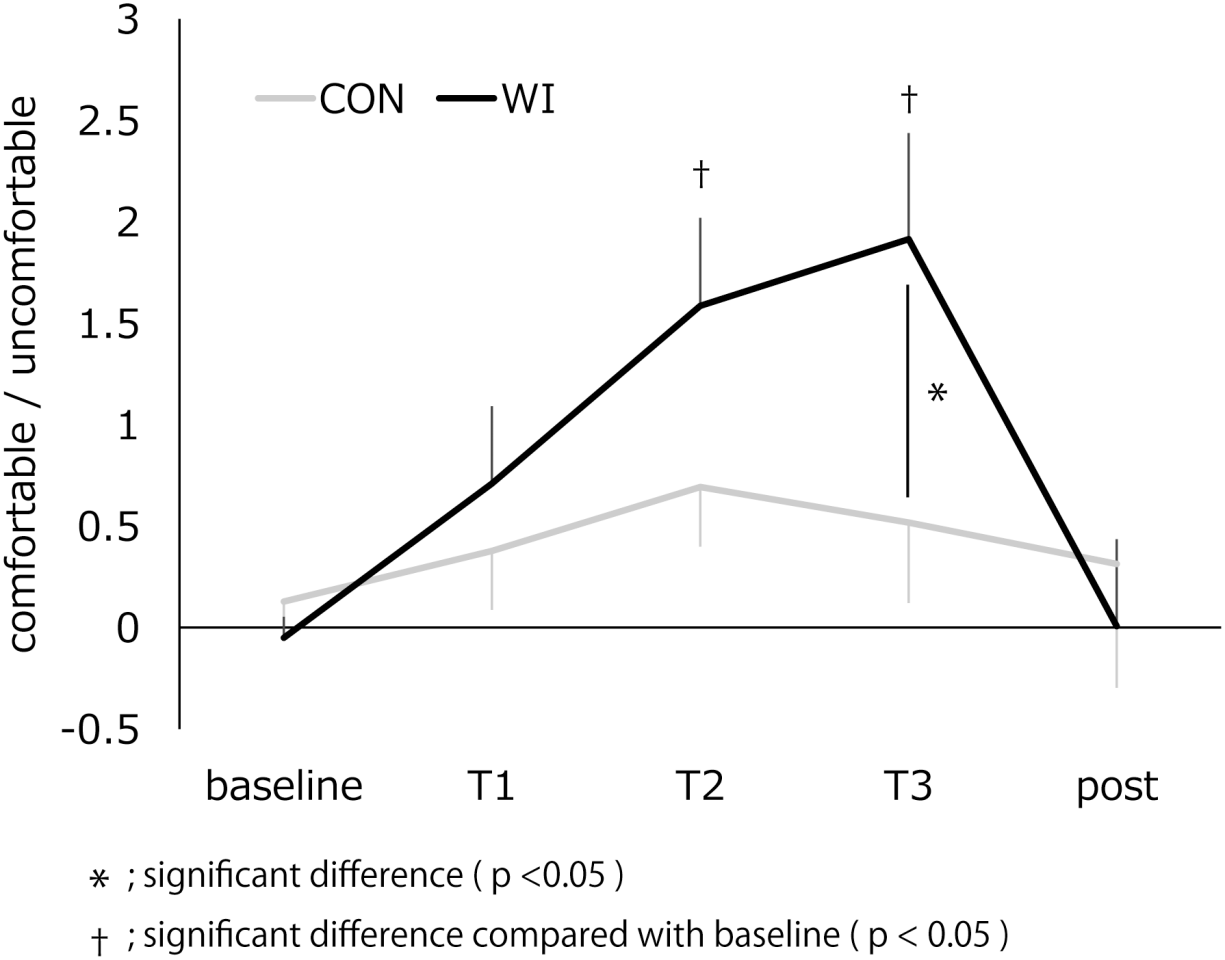
The change in visual analog scale at baseline, T1, T2, T3, and post-assessment. −5 indicated extreme uncomfortable and +5 indicated extreme comfortable. *; significant difference (p <0.05) †; significant difference compared with baseline (p < 0.05).

Fig 6 and Table 4 present the change in VAS. A repeated measures ANOVA revealed a significant interaction of “intervention” and “time” (F(4,52) = 4.069, p = 0.006). In addition, significant main effects of “time” (F(4,52) = 7.553, p = 0.000) were revealed. For WI intervention only, post hoc comparisons revealed a significantly higher value at T3 compared with baseline and post-assessment (p = 0.019 and 0.021, Bonferroni corrected), and T2 compared with baseline (p = 0.016, Bonferroni corrected). In addition, there was a significantly higher value at T3 in WI than in CON intervention (p = 0.046, Bonferroni corrected).

## Discussion

The present study examined whether water immersion changes ANS activity during a Stroop task and affects task performance. The main findings of the present study were as follows: 1) water immersion decreased the Stroop task-related sympathetic nervous response, which did not affect reaction times for Stroop task and Stroop interference; and 2) decreased SCL induced by water immersion was maintained for at least 15 min after immersion.

### Stroop task-related sympathetic nervous activity

The present results show that water immersion can decrease Stroop task-related SCR, irrespective of task difficulty, without changing reaction time and accuracy. These results indicated that water immersion intervention inhibits Stroop task-related sympathetic nervous activity. Indeed, the color–word matching Stroop task has been used in psychological stress research and was found to induce task-related sympathetic nervous activity [32]. As in the present study, Fechir et al. [32] investigated the relationship between sympathetic activation parameters, including task-related SCR, and brain activity induced in a task with variations in speed and difficulty, and found that task-related sympathetic nervous activity was related to accuracy rate. This implies that excess task-related sympathetic nervous activity has a negative effect on executive function. Animal studies have produced clear evidence that inhibition of increased sympathetic nervous activity improves cognitive function via anti-oxidant effects in the hippocampus in hypertensive rats [33].

We hypothesized that changes in ANS activity induced by water immersion would affect executive function, because previous studies have reported a relationship between Stroop interference and increased sympathetic nervous activity [34, 35]. However, we could not find an effect of water immersion on reaction time and error rate during color–word-matching Stroop task with decreasing sympathetic nervous responses. This may be because participants in the present study were healthy young adults without neurological and cardiovascular diseases that may impair executive functions. Therefore, further research of patients with high blood pressure, hypertension, and hyperlipidemia is needed.

### Effects of water immersion on autonomic nervous activity

Based on the results of SCL and ECG analyses, water immersion induced increases in parasympathetic and decreases in sympathovagal balance. Similar findings have been reported in other relevant studies that investigated the relationship between water immersion and cardiac ANS activity. One explanation for altered ANS activities during water immersion is that hydrostatic pressure mildly compresses the peripheral vasculature [36, 37], stimulating venous return [38] and baroreceptor loading [39]. Although the water temperature did not play a major role in this study, it has previously been reported to be an important parameter in water immersion-induced autonomic nervous activity. Miwa et al. [38] examined differential effects of water temperature on ANS activity during water immersion and suggested that increased core body temperature by water immersion at 40°C induced increased sympathetic nerve activity, which contrasted to the effects observed with thermoneutral water temperature (34.5°C water immersion). However, cold-water immersion causes two powerful reflexes: diving and cold shock responses known as “autonomic conflict” [1]. However, the present experimental condition with thermoneutral temperature (34°C ± 1°C) is different from either of the other two conditions. Therefore, we conclude that water immersion induced increased parasympathetic and decreased sympathovagal balance in the present study because of a hydrostatic pressure-related effect.

We found mismatching of autonomic nervous activity at post-assessment. SCL decreased significantly during water immersion, and this effect was maintained at post-assessment. While ECG spectral signals significantly changed during water immersion, they returned to baseline at post-assessment. This discrepancy provides an explanation of why SCL represents central nervous system (CNS) arousal level, whereas spectral signal directly represents cardiac sympathetic/parasympathetic nervous activity. Lim et al. [40] has shown the SCL contains substantial information that may be related to specific features of brain state and information processing. Indeed, several previous studies have demonstrated a close association of central and peripheral measures of arousal. For example, Barry et al. [41] found that the resting SCL was inversely related to alpha power in the electroencephalogram and directly related to alpha frequency. Other studies have indicated that the CNS regions responsible for generating SCL are also associated with emotional and motivational behaviors [42]. These regions include the hypothalamus and brainstem [43], amygdala [44, 45], and the orbitofrontal, cingulated, and insular cortices. Therefore, SCL has been used as a simple measure of CNS arousal. However, the results of power spectrum analysis of the ECG provide a quantitative noninvasive means of assessing the functioning of the short-term cardiovascular control systems [46]. In particular, sympathetic and parasympathetic nervous activities make frequency-specific contributions to the heart rate power spectrum, and renin–angiotensin system activity strongly modulates the amplitude of the spectral peak located at LF. Based on these reports, water immersion-induced autonomic nervous activity should be different between the heart and skin; furthermore, decreased CNS arousal level did not return to baseline level immediately after water immersion unlike cardiac autonomic nervous activity.

There were two notable limitations to the present study. First, we did not control respiratory rate during water immersion, although breathing frequency modulate ANS activity [47, 48]. Second, we did not collect tidal volume despite ventilation also modulating ANS activity [49].

### Clinical application

Water immersion and exercise have become increasingly popular, and it has been reported that these are therapeutically beneficial. Several studies have revealed that water immersion can provide relief from edema and improve blood flow [50–52]. The present results indicated that water immersion would reduce task-related sympathetic nervous activity and CNS arousal level after water immersion, suggesting that water immersion might be applicable for diagnosis induced by dysautonomia. However, our findings did not support our hypothesis that water immersion would reduce executive function evaluated by the Stroop task, which indicates that water immersion conveys little risk for falls after immersion. Further clinical research examining whether water immersion could applied as a dysautonomia diagnostic is needed.

## Conclusions

These results suggest that water immersion inhibited increased sympathetic nervous responses during executive tasks. However, the response time and error rate for executive tasks was not altered by water immersion in contrast with our hypothesis that water immersion would improve executive function induced by changes in ANS activity.
